# Structural basis for the multimerization of nonstructural protein nsp9 from SARS-CoV-2

**DOI:** 10.1186/s43556-020-00005-0

**Published:** 2020-08-20

**Authors:** Changhui Zhang, Yiping Chen, Li Li, Yan Yang, Jun He, Cheng Chen, Dan Su

**Affiliations:** 1 State Key Laboratory of Biotherapy, West China Hospital, Sichuan University, and Collaborative Innovation Center for Biotherapy, Chengdu, 610041 PR China; 2 CAS Key Laboratory of Regenerative Biology, Guangzhou Institutes of Biomedicine and Health, Chinese Academy of Sciences, Guangzhou, 510530 PR China; 3 School of Life Sciences, Tianjin University, Tianjin, 300072 PR China

**Keywords:** SARS-CoV-2, COVID-19, nsp9, Tetramer

## Abstract

Severe acute respiratory syndrome coronavirus-2 (SARS-CoV-2), the causative agent of a potentially fatal disease named coronavirus disease 2019 (COVID-19), has raised significant public health concerns globally. To date, the COVID-19 pandemic has caused millions of people to be infected with SARS-CoV-2 worldwide. It has been known since the 2003 SARS epidemic that coronaviruses (CoVs) have large RNA genomes, the replication of which requires an RNA-dependent RNA replication/transcription complex. CoV nonstructural proteins (Nsps) play pivotal roles in the assembly of this complex and associated enzymatic functions in virus genomic replication. Several smaller nonenzymatic Nsps assist with RNA-dependent RNA polymerase function. In this study, we determined the structure of SARS-CoV-2 nonstructural protein 9 (nsp9), an RNA-binding protein that is essential for CoV replication. Its homotetrameric structure with two stable dimeric interfaces provids a structural basis for understanding the mechanisms of RNA-binding protein self-assembly, which may be essential for the regulation of viral RNA replication and transcription.

## Introduction

Coronavirus disease 2019 (COVID-19), an acute respiratory distress syndrome caused by severe acute respiratory syndrome coronavirus-2 (SARS-CoV-2), is currently a global pandemic that has been spreading across all continents since late 2019 [1–3]. According to the World Health Organization COVID-19 situation reports, the number of individuals with a confirmed SARS-CoV-2 infection stood at approximately 16 million worldwide as of July 27, 2020, with related fatalities standing at over 0.64 million [4]. SARS-CoV-2 is a coronavirus (CoV) strain that belongs to the family *Coronaviridae* [5]. Genomic analysis suggests that SARS-CoV-2 is a new member of the genus *Betacoronavirus*, which is most closely related to the SARS-like virus previously identified in wild bats [6]. Human betacoronaviruses, including SARS-CoV, Middle East respiratory syndrome coronavirus (MERS-CoV), and SARS-CoV-2, share more sequence similarities with one another than with other CoVs [7]. However, SARS-CoV-2 has very high transmissibility, exhibiting rapid spreading through human-to-human contact, causing the WHO to declare it as a worldwide health emergency [8].

The genome of SARS-CoVs consists of an approximately 30-kb non-segmented positive-sense RNA sequence with a 5′ untranslated region (UTR), followed by a single open reading frame and a short flanking 3′ UTR [9]. The life cycle of SARS-CoVs starts with its binding to the cell surface receptor angiotensin-converting enzyme 2 (ACE2) through its spike glycoproteins, resulting in receptor-mediated endocytosis [10]. Upon entry into the host cell, the virus expresses its replicase gene to initially generate two precursor polyproteins, pp1a and pp1ab. These then undergo cleavage to yield 16 mature nonstructural proteins (nsp1–16) as well as several intermediate precursors by two distinct viral proteinases, the papain-like proteinase within nsp3 and a 3C-like proteinase nsp5 [11]. These nsps includes replicative enzymes (e.g., nsp12/RdRp, and nsp13/helicase), a variety of subunits performing accessory functions for viral RNA synthesis (e.g., nsp8/primase, nsp14/exoribonuclesae, and nsp15/endoribonuclease NendoU) and several smaller nonenzymatic nsps (nsp7, nsp9, and nsp10) assist with RNA-dependent RNA polymerase function [12–17].

As a member of the oligosaccharide/oligonucleotide-binding (OB-fold) superfamily, the RNA-binding protein nsp9 is critical to the RNA-dependent RNA replicase machinery of CoVs [18] nsp9 is essential for efficient viral growth as it plays a pivotal role in the formation of the replication and transcription complex machinery [19]. Mutation of the SARS-CoV *nsp9* gene through a reverse genetics approach was found to prevent the propagation of the virus [20]. The sequences of nsp9 homologs are highly conserved among betacoronaviruses especially between SARS-CoV and SARS-CoV-2 (~ 97% sequence homology), and several nsp9 homologous structures have been determined in different viral species, including SARS-CoV [19, 21–24].

To elucidate the molecular mechanisms of SARS-CoV-2 replication, which would aid in the diagnosis, treatment, and prevention of the COVID-19 pandemic, researchers have made significant efforts to characterize the Nsp structures in a short amount of time since the COVID-2019 outbreak [18]. In this paper, we report the crystal structure of SARS-CoV-2 nsp9 at 2.95 Å. This structure was revealed to be a horseshoe-like tetramer, which may play an essential role in nsp9 oligomerization and in the regulation of viral nucleic acid binding during the replication of the virus. Two significant contact surfaces stabilize the SARS-CoV-2 nsp9 tetramer. One interface is composed of a parallel association of the C-terminal alpha (α)-helices and the N-terminal loop, which are highly conserved across CoV species. Antiparallel beta (β)-strands form the other interface from both protomers of nsp9, zippering the two β-barrels together. The structure of SARS-CoV-2 nsp9 provides insights into its multimerization and the regulation of viral nucleic acid binding during CoV replication.

## Results

### Overall structure of the SARS-CoV-2 nsp9 protomer

**Fig. 1:**
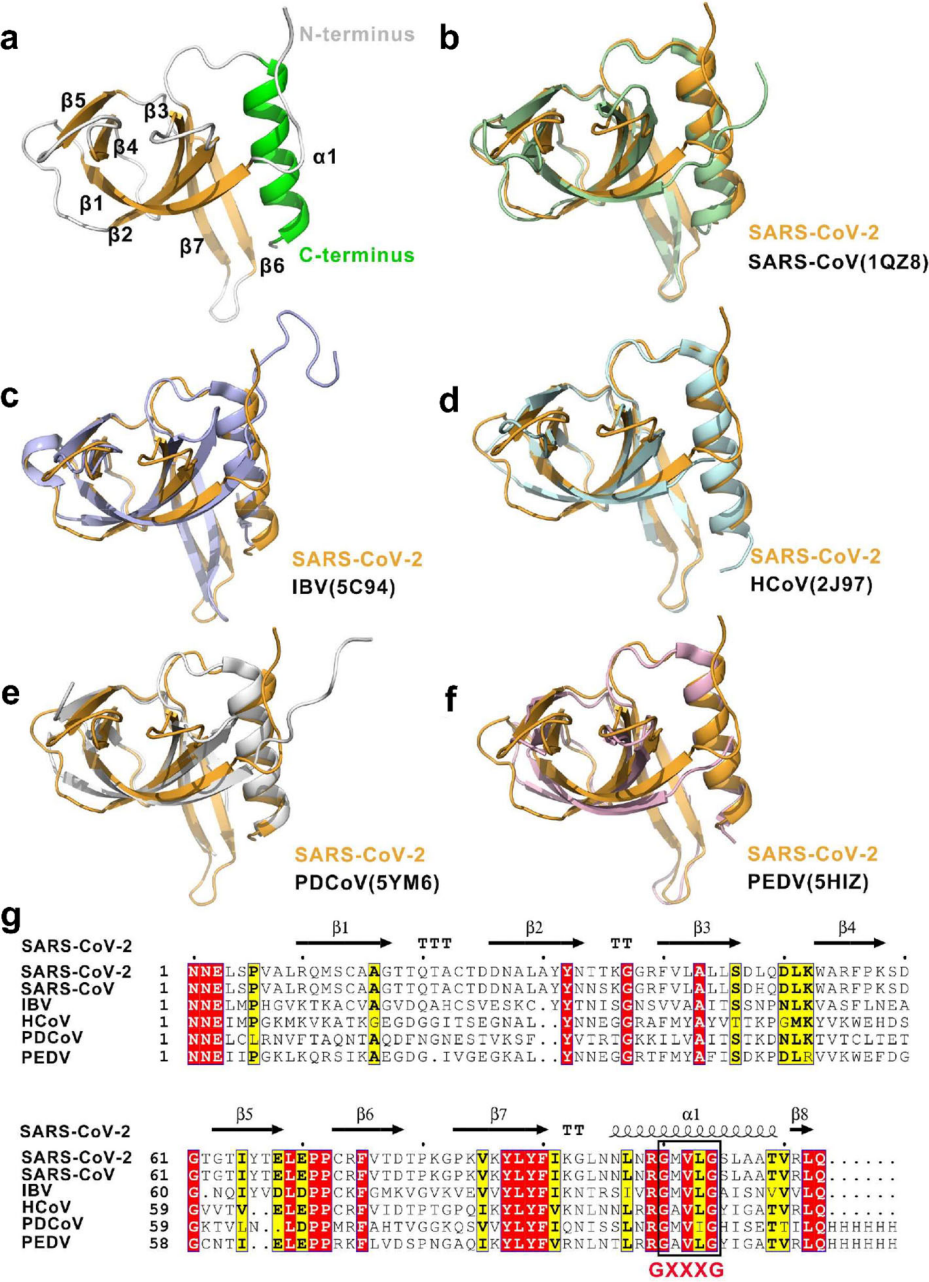
Overall structure of the SARS-CoV-2 nsp9 protomer. **a** Different views of the protomeric architecture of SARS-CoV-2 nsp9 with the secondary structure labeled. The α-helix, β-sheets, and loops are colored in green, orange, and white, respectively. **b**–**f** Superimposition of SARS-CoV-2 nsp9 with other nsp9 structures in the coronavirus family. The nsp9s from SARS-CoV-2, SARS-CoV, avian infectious bronchitis virus (IBV), human coronavirus 229E (hCoV-229E), porcine delta coronavirus (PDCoV), and porcine epidemic diarrhea virus (PDEV) are colored in orange, light blue, marine, white, and salmon, respectively. The PDB codes are indicated in the lower right corner. **g**Sequence alignment of CoV nsp9 homologs. Comparison of the SARS-CoV, IBV, HCoV, PDCoV, and PDEV sequences with the SARS-CoV-2 sequence. Identical residues are highlighted in red, and conserved residues are shown in yellow. The table was produced with ESPript 3.0, using secondary structure elements for SARS-CoV-2 nsp9 assigned using DSSP. Residues boxed in red are completely conserved

The SARS-CoV-2 nsp9 protomer is made up of seven β-strands (β_1_–β_7_) flanked by an N-terminal extension and a C-terminal α-helix (α_1_) (Fig. 1a). The core of the protein comprises a β-stranded barrel made up of two antiparallel β-sheets that are packed orthogonally. One of the sheets is formed by β_1_–β_3_ with a half strand of β_7_, and the other sheet is composed of the β_4_ and β_5_ strands. A tight β-hairpin involving β_6_ and β_7_ lies adjacent to the α-helix, extending out of the β-barrel. The elongated loops link the individual β-sheets of the barrel, along with the N-terminal β-strand and a C-terminal α-helix. The fold of this nsp9 protomer meets the structural features of OB-fold modules, one of which is a β-barrel consisting of six highly coiled antiparallel β-sheets [25]. Therefore, the structural assignment of the nsp9 monomer is the OB-fold, a compact structural motif in the OB-fold superfamily that is frequently used for nucleic acid recognition [26].

The SARS-CoV-2 nsp9 protomeric structure is highly conserved among CoVs. To date, five nsp9 structures from various CoV species have been reported; namely, SARS-CoV nsp9 (PDB: 1QZ8), avian infectious bronchitis virus (IBV) nsp9 (PDB: 5C94), human coronavirus (hCoV) 229E nsp9 (PDB: 2 J97), porcine delta coronavirus (PDCoV) nsp9 (PDB: 5YM6), and porcine epidemic diarrhea virus (PEDV) nsp9 (PDB: 5HIZ) [21–24]. A superimposition of all protomeric nsp9 backbone coordinates showed that the root-mean-square deviation values between SARS-CoV-2 and SARS-CoV, IBV, HCoV, PDCoV, and PDEV are 0.562 Å for 88 C_α_ atoms, 0.70 Å for 73 C_α_ atoms, 1.311 Å for 50 C_α_ atoms, 3.940 Å for 74 C_α_ atoms, and 0.479 Å for 52 C_α_ atoms, respectively (Fig. 1b–f). These data suggest that the nsp9 structure of SARS-Cov-2 is highly similar to that of all CoVs, especially SARS-CoV nsp9 (Fig. 1g).

### Structural basis for SARS-CoV-2 nsp9 multimerization

**Fig. 2:**
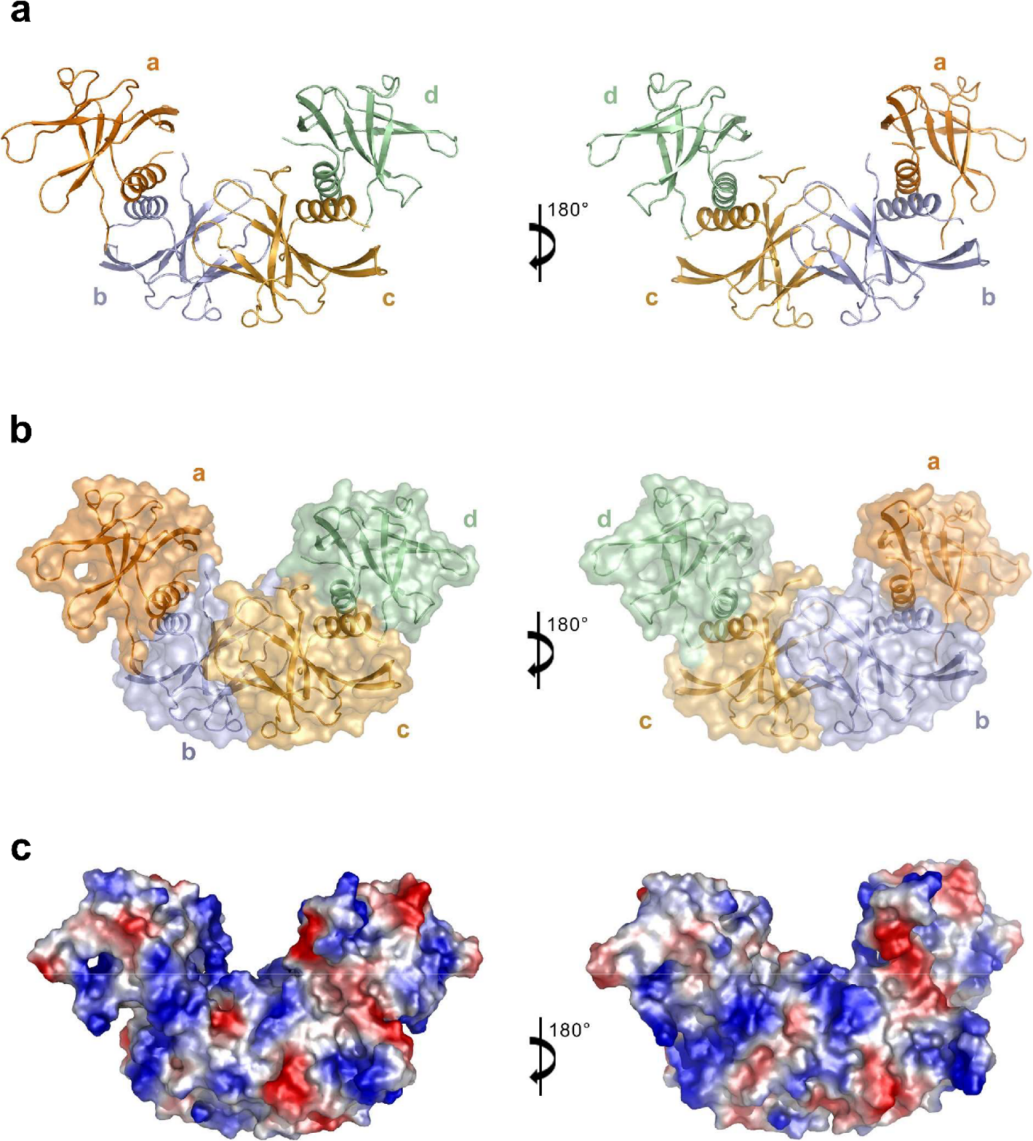
Structure of the SARS-CoV-2 nsp9 tetramer. **a** Architecture and assembly of the nsp9 tetramer. Molecules a, b, c, and d in the stable tetramer are colored in orange, light blue, bright orange and pale green, respectively, and depicted in cartoon representations. **b** The stable tetramer is colored, labeled as Fig. 2a and depicted in cartoon and surface representations. **c** The electrostatic potential surface of the stable tetramer. Blue, positive charge (+ 70 k_B_T); Red, negative charge (− 70 k_B_T)

The initial structure solved by molecular replacement showed that six SARS-CoV-2 nsp9 protomers form an OB-fold cluster in an asymmetric unit (Supplementary Fig. 1a). The OB-fold cluster is stacked in three layers (I, II, and III). For the convenience of description, we have named the protomer in layer I as molecule a’, the three protomers in layer II as molecule b’/c’/d’, and the two protomers in layer III as molecule e’/f ’. To obtain more information about the protein interfaces and the likely biological assemblies of the OB-fold cluster, we calculated the structure of SARS-CoV-2 nsp9 using PDBePISA [27]. The buried area and binding energy results (Supplementary Table 1) indicated that there is one stable tetrameric form of SARS-CoV-2 nsp9 in the crystal lattice. Each protomer associated with the tetramer assembly comes from different asymmetric units in this structure (Supplementary Fig. 1b). In order to get the best view of the tetramer of nsp9 and to provid a general description of the structure, we refined the structure to get a new asymmetry unit that contains an intact tetrameric form of nsp9. In the new asymmetric unit, four of six protomers form a stable tetramer (molecule a/b/c/d) (Fig. 2a) while the other two protomers on the side of the tetramer, which forms tetramer with other two protomers in neighbor unit. Overall, four nsp9 protomers assemble into a horseshoe-like complex, with a buried area of close to 4638 Å^2^ (Fig. 2b and c).

**Fig. 3:**
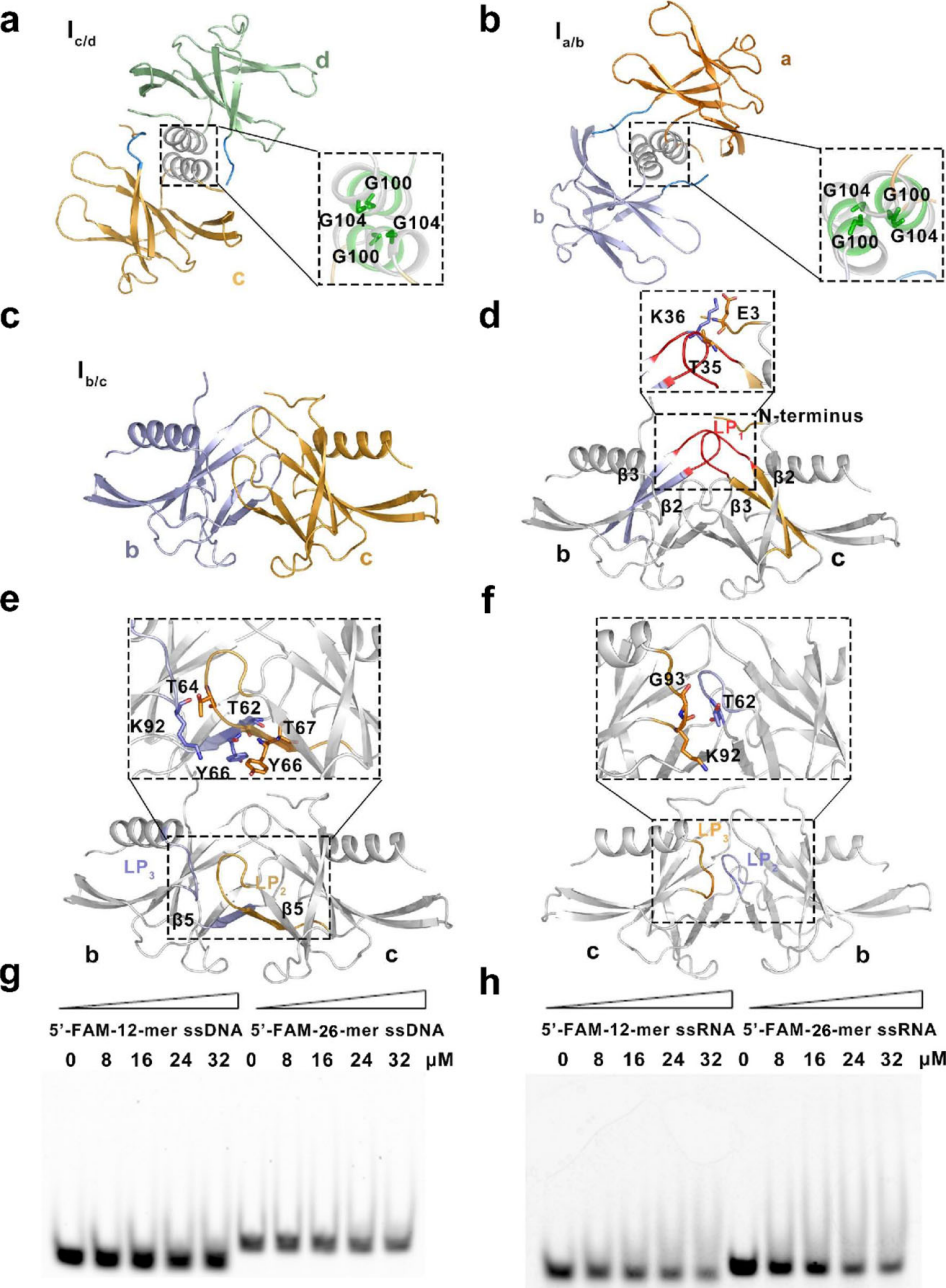
Details of structure of the SARS-CoV-2 nsp9 tetramer and function in nucleic acid binding. **a**, **b** Details of I_c/d_ and I_a/b_ in the helix interface. The molecules in these two interfaces are shown as cartoons and colored and labeled as in Fig. 2a. The expanded boxed area to the right shows a detailed view of the critical residues in the I_c/d_ and I_a/b_ interfaces, which are shown as sticks. **c** Molecules involved in I_b/c_. The molecules in this interface are shown as cartoons and colored and labeled as in Fig. 2a. **d**–**f** Detailed views of the first, second, and third contact regions in interface I_b/c_, respectively. The secondary structures of each contact region are colored and labeled according to Fig. 2a and the critical residues in each region are shown as sticks. **g** Single-stranded DNA (ssDNA)-binding abilities of SARS-CoV-2 nsp9, as determined by electrophoretic mobility shift assay. **h** Single-stranded RNA (ssRNA)-binding abilities of SARS-CoV-2 nsp9. The length of ssDNA, the state of SARS-CoV-2 nsp9, and the concentration of proteins used in the reaction system are indicated above the gel

Three interfaces (I_a/b_, I_b/c_, and I_c/d_) are engaged in the tetrameric structure in two combination modes (helix interface and sheet interface). Interfaces I_a/b_ (between molecules a and b) and I_c/d_ (between molecules c and d) share the same combination mode (i.e., the helix interface), having a buried area of 626 Å^2^ and 652 Å^2^, respectively. Each helix interface involves the conserved C-terminal α-helices (residues 96–109) that are associated in parallel and the N-terminus of nsp9 (residues 1–9) (Fig. 3a and b). The parallel α-helices allow two molecules to be packed together by a conserved protein–protein interaction motif (GXXXG). Residues G100 and G104, which are located on the helices from each subunit, play a pivotal role in tetramer stabilization. Further stabilization of this tetrameric interface is given by residues 6–10 in the N-terminus, clipping to the edge of β_6_ from its neighboring partner.

Compared with previously reported CoV nsp9 structures, interface I_b/c_ (between molecules b and c) is a unique binding surface located in the center of the SARS-CoV-2 nsp9 tetrameric structure (Fig. 3c). This interactive region adopts a sheet-interface style formed by β_5_ (residues 64–68) and three connection loops (LP_1_, LP_2_, and LP_3_) from both molecules b and c. The buried area of the I_b/c_ interface is 1040 Å^2^, which is the largest buried area compared with those of interfaces I_c/d_ and I_a/b_ in the SARS-CoV-2 nsp9 tetramer. There are three major contact regions in the I_b/c_ interface. The first contact region associates with two LP_1_ loops (residues 33–42), connecting β_2_ and β_3_ from each of the subunits of c and b. Part of the N-terminal tail (residues 2–5) from molecule c is also involved in this contact surface. Two hydrogen bonds and one salt bond are involved in the interface between residues E3 and T35 in molecule c and residue K36 in molecular b (Fig. 3d). Additionally, residues G38, F40, V41, and L44 make a significant contribution to this hydrophobic contact surface. The second contact region consists of connecting LP_2_ (residues 58–63) and LP_3_ loops (residues 91–96) and β_5_ (residues 64–68) from each of the subunits. The LP_2_ loop on molecule c comes in direct contact with LP_3_ on molecule b. An antiparallel β-sheet located at the center of the sheet interface is composed of two β_5_ strands. In this hydrophobic contact surface, three residues (T62, T64, and Y66) on molecule c form four hydrogen bonds with residues K92, T67, and Y66 on molecule b, respectively (Fig. 3e). The third contact region involved in interface I_b/c_ is composed of the LP_3_ loop on molecule c and the LP_2_ loop on molecule b. Residues K92 and G93 on molecule c form two hydrogen bonds with residue T62 on molecule b (Fig. 3f). These three contact surfaces in interface I_b/c_ contribute a hydrophobic base with eight hydrogen bonds and one salt bridge, making the SARS-CoV-2 nsp9 tetramer extremely stable in the crystal structure.

### SARS-CoV-2 nsp9 with nucleic acid-binding ability

Our previous research on nsp9 from IBV showed that the multimerization of this protein was necessary for protein–nucleic acid interaction [23]. In this present study, we observed the nucleic acid-binding ability of SARS-CoV-2 nsp9, using the electrophoretic mobility shift assay. Under favorable conditions, the nsp9 wild type was incubated with single-stranded nucleic acids. As shown in Fig. 3g and h, the free single-stranded DNA and RNA bands in the lanes associated with nsp9 were slightly reduced with the increasing concentration of nsp9. Additionally, we observed the ability of nsp9 to interact with a range of sizes and types of single-stranded nucleic acids. Based on these results, we can conclude that nsp9 prefers to interact with the single-stranded nucleic acid.

## Discussion

In this study, we first revealed the horseshoe-like tetrameric structure of nsp9 encoded by the genomic RNA of SARS-CoV-2. This homotetrameric structure comprised two significant styles of dimeric conformation that were found in nsp9 structures published in the last decade [19, 21]. These structural results from independent research groups revealed that the dimerization of nsp9 might occur biologically and could play a pivotal role in the nucleic acid-binding function of the protein. However, because the dimerized nsp9 structure involves different dimeric forms, an interesting question is which of these is the major unit that interacts with nucleic acids. In the horseshoe-like tetrameric structure of SARS-CoV-2 nsp9, we found that two dimeric forms exist. One of them, named the helix interface and first reported by Egloff et al. (2004) [21], is organized by the parallel association of the C-terminal α-helix and the N-terminal loop. The second dimeric form, named the sheet interface and first mentioned by Sutton et al. (2004) [19], is formed by the zippering of each β_5_ strand from both subunits. The sheet interface is much more stable in the tetrameric structure of SARS-CoV-2 nsp9 than in SARS-CoV nsp9. Aside from the β5 strand, there are three loops (LP_1_, LP_2_, and LP_3_) involved in the nsp9 tetrameric interface. Therefore, we propose that these two dimeric forms are the fundamental units for building the high-order oligomers of nsp9. According to two different research groups that studied nsp9 mutants, the mutation of conserved residues located on the C-terminal α-helix (e.g., SARS-CoV nsp9 G104E (PDB: 3EE7)) or the unexpected appearance of a disulfide on β_5_ (hCoV-229E nsp9 wild type (PDB: 2 J97)) could significantly change the dimer interface of nsp9 and its ability to bind with DNA or RNA [20, 22]. In summary, the two dimeric interfaces that occurred in all crystal structures of nsp9 are reasonable and likely contribute to the formation of the higher oligomeric states of the protein.

## Materials and methods

### Plasmid construction, protein expression, and purification

The SARS-CoV-2 *nsp9* gene (genome nucleotides 12,686–13,024; Gene ID: 43740578) was synthesized and cloned into the pET-28a (+)-SUMO vector using BamHI and XhoI sites (Qingke Biotech, China). The recombinant plasmids, which were confirmed by DNA sequencing (Sangon Biotech, China), were then used to transform *Escherichia coli* BL21 (DE3) (Transgene, China). Cells were cultured in Luria broth medium at 37 °C until the optical density at 600 nm reached 0.6–0.8. Protein expression was then induced by incubating the cells with 0.5 mM isopropyl β-d-1-thiogalactopyranoside at 16 °C for 18 h. The nsp9 protein was labeled with a His-SUMO tag with a Ulp1 cleavage site, purified by nickel-nitrilotriacetic acid affinity chromatography (Qiagen, Germany), and then cleaved with Ulp1 protease. The cleaved and tag-removed protein was loaded onto a Resource S chromatography column (GE Healthcare, USA) and eluted with a linear gradient of 10 mM to 1 M NaCl. The peak fraction containing the target protein was pooled, then concentrated to 1 mL using Amicon Ultra concentrators (cutoff size of 10 kDa; Millipore, USA), and finally loaded onto a Superdex 200 column (GE Healthcare, USA) for further purification with a buffer composed of 20 mM Tris (pH 8.0), 150 mM NaCl, 1 mM dithiothreitol, and 10% glycerol (v/v). The purity of the proteins was greater than 95%, as confirmed by sodium dodecyl sulfate polyacrylamide gel electrophoresis (Supplementary Fig. 2a and 2b).

### Protein crystallization and optimization

Using the hanging-drop vapor diffusion method, the nsp9 protein was quantified using absorbance readings at A280 nm (Thermo NanoDrop 2000, USA) and concentrated to 17 mg/mL for the crystallization trials, by mixing 1 μL of protein with 1 μL of reservoir solution at 20 °C. Commercial crystallization kits (Hampton Research, UK) were used to obtain the initial crystallization conditions. The protein crystals were obtained in a solution containing 100 mM citric acid (pH 3.0), 200 mM potassium sodium tartrate tetrahydrate, and 2.0 M (NH_4_)_2_SO_4_ after growth for 2 days at 20 °C (Supplementary Fig. 2c and 2d). The crystals were then transferred to a solution containing 100 mM citric acid (pH 3.0), 200 mM sodium metformin, and 2.5 M (NH_4_)_2_SO_4_, and the mixture was then frozen and stored in liquid nitrogen for future data collection.

### Data collection, processing, and structure determination

The nsp9 crystal diffraction data were collected at − 196 °C, using the SSRF Beamline BL18U1 apparatus (Shanghai, China) at a wavelength of 0.97930 Å. Data were processed and scaled using the HKL2000 package (Supplementary Fig. 2e) [28]. The nsp9 structure was solved by Phaser in the CCP4 program suite using molecular replacement with the structure of SARS-CoV nsp9 RNA-replicase (PDB: 1QZ8) as an initial search model [29]. Cycles of refinement and model building were carried out using the REFMAC5, Phenix, and COOT software programs [30, 31]. Model geometry was verified using MolProbity. The single crystals of SARS-CoV-2 nsp9 are in space group *C222*_1_, with cell dimensions of *a* = 88.7 Å, *b* = 134.5 Å, *c* = 167.0 Å, and ɑ = β = γ = 90°. Both the Matthews coefficient estimation and the self-rotation function suggested the presence of six molecules per asymmetric unit. The final structure was refined to 2.95 Å resolution. The final R_work_ and R_free_ for the refined structure were 21.3% and 29.9%, respectively. In total, 96.1% of the amino acid residues fell in the most-favored region and the additionally allowed region of the Ramachandran plot. The structural figures were drawn using PyMOL [32]. The data collection and refinement statistics are shown in Table 1.

**Table 1:** SARS-CoV-2 nsp9 data collection and refinement statistics

	SARS-CoV-2 nsp9
***Data collection***	
Resolution (Å)	2.95
Space group	*C222*_1_
Unit-cell parameters (Å, °)	*a =* 88.7*, b =* 134.5 *c =* 167.0, *α = β = γ =* 90
Resolution (Å)	50.00–2.95 (3.04–2.95)
R_merge_^a^ (%)	22.1 (59.3)
R_*pim*_^b^ (%)	6.6 (17.0)
Average *I/σ(I)*	11.6 (4.5)
No. of observed reflections	40,346 (3400)
No. of unique reflections	21,270 (1762)
Completeness (%)	99.55 (97.3)
Multiplicity	12.4 (2.3)
Matthews coefficient (Å^3^Da^−1^)	3.26
Solvent content (%)	62.3
Molecules per asymmetric unit	6
***Refinement***	
Resolution (Å)	44.49–2.95
R_work_/R_free_	0.21/0.30
Ramachandran favored (%)	88.31
Ramachandran outliers (%)	3.32
***No. of atoms***	
Protein	4963
Water	66
Wilson B value	87.32
***Root-mean-square deviations***	
Bond length (Å)	0.010
Bond angle (°)	1.293

^a^*R*_merge_ = ∑_*hkl*_∑_*i*_ ∣ *I*_*i*_(*hkl*) – 〈*I*(*hkl*)〉 ∣ /∑_*hkl*_∑_*i*_*I*_*i*_(*hkl*), where *I*_*i*_(*hkl*) is an individual intensity measurement and 〈*I*(*hkl*)〉 is the average intensity for all *i* reflections

^b^*R*_*pim*_ is approximately estimated by multiplying the R_merge_ value by the factor [1/(*N* − 1)]^1/2^, where *N* is the overall redundancy of the data set

### Electrophoretic mobility shift assay

The 5′-FAM-labeled 12-mer single-stranded DNA oligonucleotide 5′-GCTTTGATTTCG-3′, 5′-FAM-labeled 26-mer single-stranded DNA oligonucleotide 5′-GCTTTGATTTCGTGCATCTATGGAGC-3′, 5′-FAM-labeled 12-mer single-stranded RNA oligonucleotide 5′-GCUUUGAUUUCG-3′ and 5′-FAM-labeled 26-mer single-stranded RNA oligonucleotide 5′-GCUUUGAUUUCGUGCAUCUAUGGAGC-3′ were used for the electrophoretic mobility shift assay. Initially, 2 nmol DNA was incubated with different concentrations of nsp9 protein (in the native), in a solution containing 10 mM 4-(2-hydroxyethyl)-1-piperazineethanesulfonic acid (pH 8.0), 50 mM KCl, 1 mM ethylenediaminetetraacetic acid (EDTA), 0.05% Triton-X-100, and 5% glycerol, for 30 min at 4 °C. Thereafter, a 10× loading buffer (250 mM Tris-HCl (pH 7.9) and 40% glycerol) was added to the mixture. Then, samples were electrophoresed on 6.5% non-denaturing Tris–borate–EDTA (TBE) polyacrylamide gels for 40 min at a voltage of 100 V, and the results were determined with a ChemiDoc Touch Imaging system (Bio-Rad, USA).

### Protein data Bank accession codes

The structure factors and atomic coordinates have been deposited in the Protein Data Bank under the PDB ID codes 7BWQ.

## Supplementary information

**Additional file 1: Figure S1.** The OB-fold cluster of SARS-CoV-2 nsp9. (a) The different layers are colored orange, light blue, and pale green, respectively, and protomers in every layer are depicted in cartoon representations and labeled with uppercase letters. (b) Molecules d’, c’, a_sym_, and b_sym_ in the stable tetramer are colored in bright orange, pale green, light blue, and orange, respectively, and depicted in cartoon and surface representations.

**Additional file 2: Figure S2.** Purification and X-ray data collection of SARS-CoV-2 nsp9. (a) The chromatography of the purification of SARS-CoV-1 nsp9 with a Superdex200™ 10/300 column. (b) SDS-PAGE analysis result of SARS-CoV-2 nsp9. M is the protein marker. (c) The primary crystal state of SARS-CoV-2 nsp9 in the commercial crystallization kit. (d) The crystal for X-ray data collection of SARS-CoV-2 nsp9 after crystallization condition optimized. (e) X-ray diffraction pattern for structure analysis (left) and X-ray diffraction point atlas of SARS-CoV-2 nsp9 (right).

**Additional file 3: Table S1.** PDBePISA calculation of interface parameters of the quaternary structure of SARS-CoV-2 nsp9.

## Data Availability

All data generated or analysed during this study are included in this published article [and its supplementary information files].

## Abbreviations

CoV: Coronavirus
COVID-19: Coronavirus disease 2019
hCoV: Human coronavirus
LP: Loop protein
OB-Fold superfamily: oligosaccharide/oligonucleotide-binding superfamily
nsp9: Nonstructural protein 9
SARS-CoV-2: Severe acute respiratory syndrome coronavirus-2
