# Supplementary figures and images for: Structural basis for the multimerization of nonstructural protein nsp9 from SARS-CoV-2
Source: Mol Biomed. 2020 Aug 20;1:5. doi: 10.1186/s43556-020-00005-0 (PMC7438161; doi:10.1186/s43556-020-00005-0)

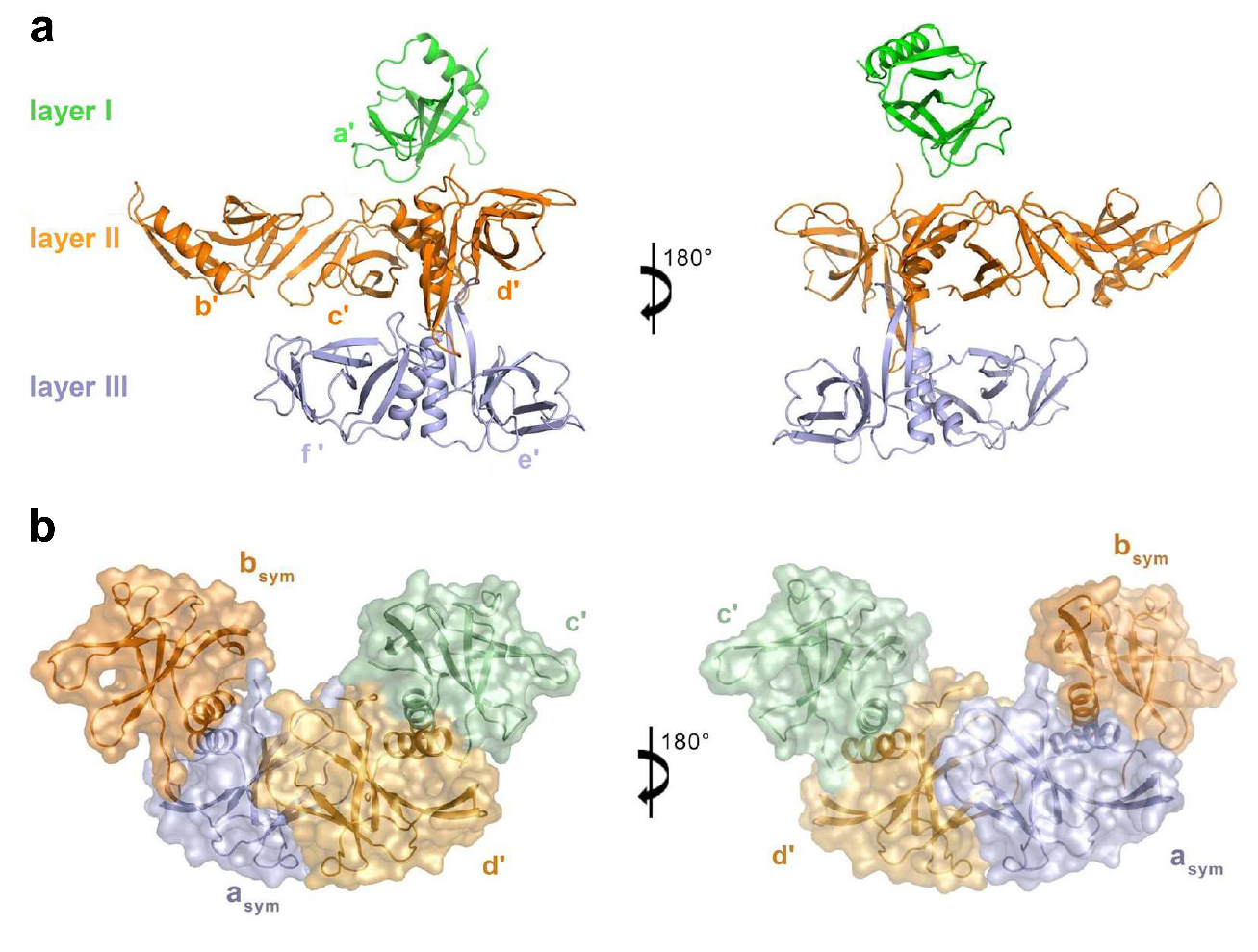
**Supplementary Fig. 1**

Supplement: Supplementary file 1 — Additional file 1: Figure S1. The OB-fold cluster of SARS-CoV-2 nsp9. (a) The different layers are colored orange, light blue, and pale green, respectively, and protomers in every layer are depicted in cartoon representations and labeled with uppercase letters. (b) Molecules d’, c’, asym, and bsym in the stable tetramer are colored in bright orange, pale green, light blue, and orange, respectively, and depicted in cartoon and surface representations. [file 43556_2020_5_MOESM1_ESM.docx]

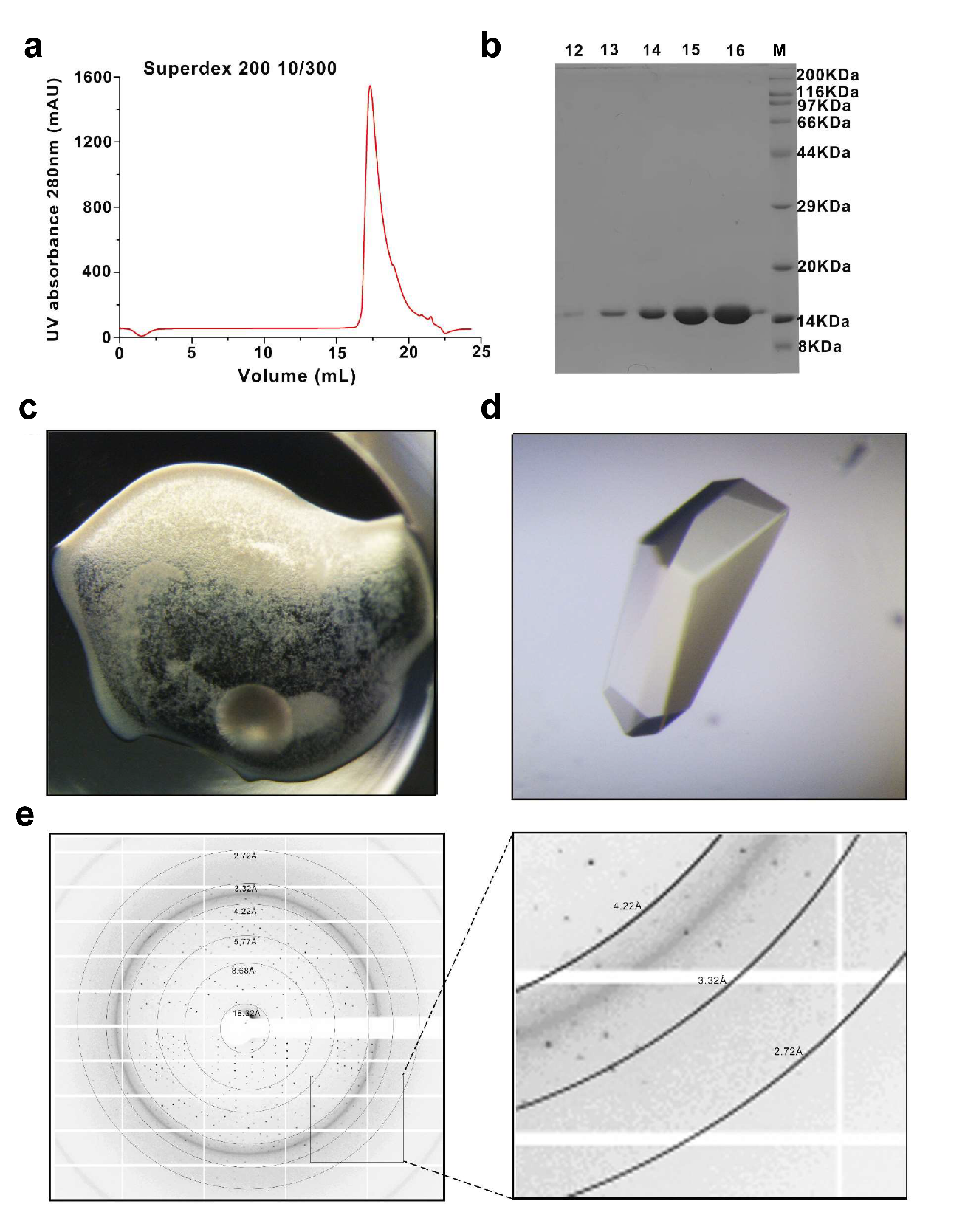


**Supplementary Fig. 2**

Supplement: Supplementary file 2 — Additional file 2: Figure S2. Purification and X-ray data collection of SARS-CoV-2 nsp9. (a) The chromatography of the purification of SARS-CoV-1 nsp9 with a Superdex200™ 10/300 column. (b) SDS-PAGE analysis result of SARS-CoV-2 nsp9. M is the protein marker. (c) The primary crystal state of SARS-CoV-2 nsp9 in the commercial crystallization kit. (d) The crystal for X-ray data collection of SARS-CoV-2 nsp9 after crystallization condition optimized. (e) X-ray diffraction pattern for structure analysis (left) and X-ray diffraction point atlas of SARS-CoV-2 nsp9 (right). [file 43556_2020_5_MOESM2_ESM.docx]
