# Supplementary material for: Structural basis for the multimerization of nonstructural protein nsp9 from SARS-CoV-2
Source: Mol Biomed. 2020 Aug 20;1:5. doi: 10.1186/s43556-020-00005-0 (PMC7438161; doi:10.1186/s43556-020-00005-0)
Supplement: Supplementary file 3 — Additional file 3: Table S1. PDBePISA calculation of interface parameters of the quaternary structure of SARS-CoV-2 nsp9. [file 43556_2020_5_MOESM3_ESM.docx]

**Supplementary Table. 1 PDBePISA calculation of interface parameters of the quaternary structure of SARS-CoV-2 nsp9.**

| **PQS set**  **NN.** | **Size (mm)** | **Formula** | **Composition** | **Id** | **Stable** | **Surface**  **area (sq. Å)** | **Buried**  **area (sq. Å)** | **Δ*G*_int_**  **(kcal/mol)** | **Δ*G*_diss_**  **(kcal/mol)** |
| --- | --- | --- | --- | --- | --- | --- | --- | --- | --- |
| 1 (*) | 2 | A2 | e'f ' | 1 | Yes | 11,920 | 1310 | -14.4 | 3.7 |
|  | 2 | A2 | a_sym_b_sym_ | 1 | Yes | 11,970 | 1300 | -14.3 | 3.7 |
|  | 2 | A2 | c'd' | 1 | Yes | 11,880 | 1250 | -14.4 | 3.2 |
| **2 (*)** | **4** | **A4** | **a_sym_b_sym_c'd'** | **2** | **Yes** | **21,770** | **4640** | **-39.0** | **1.9** |
|  | 2 | A2 | e'f ' | 1 | Yes | 11,920 | 1310 | -14.4 | 3.7 |
| 3 (*) | 2 | A2 | a_sym_d' | 3 | Yes | 11,150 | 2080 | -10.3 | 2.7 |
|  | 1 | A | b' | 4 | Yes | 6790 | 0 | 0.0 | -0.0 |
|  | 1 | A | c' | 4 | Yes | 6390 | 0 | 0.0 | -0.0 |
|  | 1 | A | e' | 4 | Yes | 6450 | 0 | 0.0 | -0.0 |
|  | 1 | A | f ' | 4 | Yes | 6780 | 0 | 0.0 | -0.0 |

The stable tetramer is in bold font for emphasis.

Δ*G*_int_, intrinsic free energy change; Δ*G*_diss_, free energy change upon dissociation.
